# Exploitation of inland salt lake water by dilution and nutrient enrichment to cultivate *Vischeria* sp. WL1 (Eustigmatophyceae) for biomass and oil production

**DOI:** 10.1016/j.btre.2023.e00823

**Published:** 2023-12-11

**Authors:** Xiang Gao, Xin jing, Jiahong Li, Min Guo, Lei Liu, Zhengke Li, Kaihui Liu, Derui Zhu

**Affiliations:** a School of Food and Biological Engineering, Shaanxi University of Science & Technology, Xi'an, Shaanxi 710021, China; b Research Center of Basic Medical Science, Medical College, Qinghai University, Xining, Qinghai 810016, China

**Keywords:** Microalgae, Mineral nutrients, Oil production, Salt lake water, Sustainable resource

## Abstract

Salt lakes are significant components of global inland waters. Salt lake (SL) water can provide precious mineral resource for microbial growth. The prospect of utilizing diluted SL water for cultivation of a terrestrial oil-producing microalga *Vischeria* sp. WL1 was evaluated under laboratory conditions. Based on the detected mineral element composition, the water from Gouchi Salt Lake was diluted 2, 4, 6 and 8 folds and used with supplementation of additional nitrogen, phosphorus and iron (SL^+^ water). It was found that 4 folds diluted SL^+^ water was most favorable for biomass and oil production. When cultivated in this condition, *Vischeria* sp. WL1 gained a biomass yield of 0.82 g L^−1^ and an oil yield of 0.56 g L^−1^ after 24 days of cultivation, which is comparable to the optimum productivity we previously established. In addition, total monounsaturated fatty acid contents (64.4∼68.1 %) of the oils resulted from cultures in diluted SL^+^waters were higher than that in the control (55.5 %). It was also noteworthy that in all these cultures the oil contents (652.0∼681.0 mg g^−1^) accounted for the most of the biomass, which are far more than the protein and starch contents. This study demonstrates the feasibility of using SL water as a cost-effective mineral resource to cultivate microalgae for biomass and oil production.

## Highlights

• Diluted salt lake water with nutrient addition (SL^+^) was used for algal cultivation.
• Cell size and pigment contents in the cultures were changed in diluted SL^+^ waters.
• Oil yield reached 0.56 g L^−1^ after 24 days cultivation in 4-folds diluted SL^+^ water.
• The starch content was continuously increased during the whole cultivation.

## Graphical abstract

**Figure:**
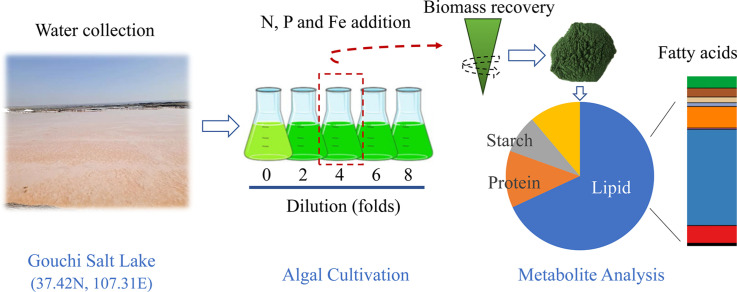

## 1 Introduction

Salt lakes are widely distributed and commonly exist in semi-arid and arid regions. Large salt lakes represent 44 % of the volume and 23 % of the area of all lakes on Earth [1]. Salt lakes are rich in natural resources, such as salt deposited resources, brine resources and biological resources. The common ions in salt lakes are calcium ion, magnesium ion, sodium ion, potassium ion, chloride ion, sulfate radical ion, carbonate ion and bicarbonate ion [2]. These rich mineral elements support halophilic microorganisms that have evolved the capability to tolerate high salinity [3,4]. Salt lake (SL) water may also serve as a cheap mineral resource for cultivation of the microorganisms that are not derived from salt lakes. Thus far, related technologies of utilizing SL water for microbial cultivation have not been fully developed.

Microalgae are an important group of photoautotrophic microorganisms that live in marine, freshwater and soil ecosystems. They can produce and accumulate value-added biochemicals, such as oils, pigments, proteins and carbohydrates [5], [6], [7], [8], [9]. The microalgae that grow in salt lakes, such as *Tetraselmis* and *Dunaliella* strains, have been isolated for producing glycerol and oil [10,11]. They are highly salt-tolerant strains and can be cultivated with SL water directly. However, for less salt-tolerant and salt-sensitive microalgal strains, how to utilize SL water for cultivation and thus reduce the consumption of industrial mineral resource is an important issue. The nutritional demand (mainly mineral elements) required for microalgae cultivation represents approximately 10–15 % of the total cost of biomass production [12]. Estimated with the usually used BG11 medium [13], the consumption of mineral elements in 30 ton water may need 22,500 CNY (Chinese yuan). Previously, seawater has been suggested as a good solution for saving freshwater resource and the cost for mineral elements [14,15]. However, in a place far from the sea or in inland regions, it is not economically practical. Moreover, the mineral nutrient compositions in various SL waters are very different [16], which may pose different or even adverse effects on the growth of microalgae. Thus, microalgal cultivation with SL water is a promising but challenging task.

Eustigmatophyceae are a distinct lineage of stramenopile algae with a relatively small number (∼30) of described species [17]. The eustigmatophytes are known for their capability to accumulate a large amount of oils with high content in polyunsaturated fatty acids (PUFAs) and thus serve as important candidates for the development of biofuels and value-added foods [18], [19], [20]. Some marine and freshwater eustigmatophytes, including *Nannochloropsis* spp., *Eustigmatos* cf. *polyphem, Vischeria stellata* and *Vischeria punctata* have been isolated and utilized for oil production [21], [22], [23], [24]. However, terrestrial strains have been rarely exploited. Recently, we isolated an oil-producing eustigmatophyte, *Vischeria* sp. WL1, from a dryland biocrust and assessed its oil production capability [8]. Unlike freshwater microalgal strains, this terrestrial strain exhibited salt tolerance, and supplementation of 0.3 M NaCl in cultural medium could result in a cost-effective production of oils [8]. Thus, we hypothesize that *Vischeria* sp. WL1 may be a suitable strain to be cultivated by SL water, thereby saving the cost in mineral elements.

The present study aimed to evaluate the growth of *Vischeria* sp. WL1 in diluted SL water augmented with essential nutrients, to determine the feasibility of using SL water as a main nutritional source. Meanwhile, the accumulation of protein, starch and oil in the culture or biomass was evaluated, with the focus on oil production.

## 2 Materials and methods

### 2.1 Microorganism and growth condition

*Vischeria* sp. WL1 was isolated from a biocrust in the arid steppe of western China and was identified by morphological and molecular analyses [8]. The inoculum was regularly maintained in BG11 medium [13] at 25 ℃ under continuous illumination of 60 μmol photons m^−2^ s^−1^ (LED white light). The optimum medium (BG11m) for its oil production was BG11_0_ (NaNO_3_-deprived BG11) supplemented with 4.5 mM NaNO_3_ [8], which was used as a control for comparison in this study. Cultural experiments were carried out in 250 mL glass flasks containing 100 mL of medium on a rotary shaker (120 rpm).

### 2.2 SL water, dilution and element addition

SL water was sampled from Gouchi Salt Lake in Dingbian County, Shaanxi Province, China (37.42.51 N, 107.31.15E). The pH of the SL water was 7.57. The SL water was suction filtered with filter paper (15–20 μm in aperture) and then filtered with 0.45 μm membrane filter. The filtered water was stored at 4 ℃. The element composition of the SL water was analyzed at Qinghai Provincial Center for Disease Control and Prevention, China (Table 1). The filtered SL water were diluted 2, 4, 6 and 8 folds with sterile water. Prior to algal cultivation, the non-diluted and diluted SL waters were nutritionally augmented with 4.5 mM NaNO_3_, 0.23 mM K_2_HPO_4_ and 16.3 μM EDTA-Fe^2+^ (their final concentrations being equivalent to those in BG11m medium).

**Table 1:** The element composition of the filtered Gouchi Salt Lake water.

Compound	Content (g L^−1^ or *mg L^−1^)	Compound	Content (*mg L^−1^ or ^#^µg L^−1^)
Total dissolved solids	273.8	Bromine	8.38*
Chloride	132.9	Carbonate	0.50*
Sulfate	64.2	Bicarbonate	0.12*
Chlorine	5.77	Calcium	0.86*
Sulfur	0.45	Boron	0.34*
Sodium	0.43	Phosphorus	0.09*
Potassium	0.35	Manganese	8.01^#^
Magnesium	0.20	Molybdenum	1.58^#^
Fluoride	36.3*	Iron	0.65^#^
Nitrate nitrogen	36.9*	Zinc	0.28^#^
Nitrite nitrogen	<0.001*	Copper	0.23^#^
Metasillicio acid	24.3*	Cobalt	0.004^#^

### 2.3 Growth and biomass determination

Algal growth and biomass were determined as described [5,25] with slight modifications. In brief, growth was evaluated by measuring the optical density at 750 nm (OD_750_) in a spectrophotometer (SP-1920, Spectrum Instruments, China) every 4 days. Meanwhile, each of 20 mL algal culture was collected and centrifuged at 1776 × *g* for 5 min. The pellet was freeze-dried until a constant weight was achieved and weighed. The biomass concentration was expressed as dry weight/volume (g L^−1^).

### 2.4 Analysis of cell size, chlorophyll a and carotenoid contents

Each of 10 μL algal culture was subjected to observation under an optical microscope (VIYEE WYS-08C, China) and cell size was measured using ImageJ [26]. For determining chlorophyll a and carotenoid contents, 1 mL algal culture was collected by centrifugation (6000 × *g*, 5 min) and then suspended in 1 ml methanol. The suspension was crushed by steel beads (180 rpm, 10 min) in a high-throughput tissue homogenizer (Wonbio-L, Shanghai Wonbio Biotechnology Co., Ltd, China). After centrifugation (6000 × *g*, 10 min), the supernatant was measured at 665.2, 470 and 652.4 nm using the above spectrophotometer. The chlorophyll a (Chl a) and carotenoid concentrations were calculated using the following equations [27]: (1) Chl a (μg ml^−1^) = 16.72×A_665__.2_–9.16×A_652.4_; (2) Carotenoid (μg ml^−1^) = (1000×A_470_–1.63×Chl a)/221.

### 2.5 Analysis of oil production and fatty acid composition

The fluorescent dye, Nile red, is the commonly used for oil content detection in microalgal cells [28,29]. For intracellular oil observation, algal cells were suspended in 20 % methyl sulfoxide solution and maintained at 45 ℃ in a water bath for 30 min, followed by dyeing with 0.1 mg mL^−1^ Nile red acetone solution in darkness for 20 min. After centrifugation, the pelleted cells were washed with deionized water to remove the unfixed dye. The fluorescence signal (575 nm) in single dyed cells were observed by a laser confocal fluorescence microscopy (Zeiss LSM 510-META, Germany) with the excitation at 480 nm.

For tracing the change of oil content during cultivation, the total fluorescence intensity of algal cell oils was detected with a fluorescence spectrophotometer (930 N, Zhuhai Dshing Company, China) with the excitation at 480 nm and emission at 585 nm. In brief, 1 mL algal culture was collected and crushed by the aforementioned tissue homogenizer. The broken cells were centrifuged and then the supernatant was incubated with 20 μL Nile red acetone solution for dyeing. The relative fluorescence intensity of Nile red in the sample (A.U. mL^−1^) was obtained after subtracting the fluorescence intensity of Nile red alone in the solution from the fluorescence intensity of stained oils. The oil extraction, absolute quantification, and fatty acid composition analysis were conducted as previously described [8].

### 2.6 Determination of protein and starch contents

Cells of 1 mL algal culture were broken by the high-throughput tissue homogenizer as above-mentioned. The supernatant was collected after centrifugation and subjected to protein and starch determination. Total protein was measured using the Coomassie brilliant blue staining method [30]. The starch content was measured using the phenol-sulfuric acid method [31].

### 2.7 Statistical analysis

For each experiment, data presented are the mean of three independent replicates. The data were evaluated using the one-way ANOVA in SPSS ver. 26 with Tukey's multiple comparison test. Significant levels were set to *P* < 0.05.

## 3 Results and discussion

### 3.1 Cell growth and biomass production during cultivation

The water from Gouchi Salt Lake is rich in chloride, sulfate, sodium, potassium and magnesium, but lack of nitrogen, phosphorus, calcium and iron (Table 1). Particularly, the molar concentrations of Cl^−1^ and SO_4_^2−^ are approximately 500 and 70 folds of those in BG11m medium. Compared to seawater [14,15], the calcium concentration in this SL water is much lower. Nitrogen and phosphorus are the important macro-elements for microalgae growth [32]; Iron is an essential micro-element that functions in many biological processes of microalgae [33]. In a previous test, *Vischeria* sp. WL1 cultivated in the SL water (either diluted or non-diluted) without supply of additional nitrogen, phosphorus and iron showed a very slow growth (data not shown). Thus in the following experiments, the three elements were supplemented in the SL water (hereinafter referred to as SL^+^water) as indicated in the Methods.

**Fig. 1:**
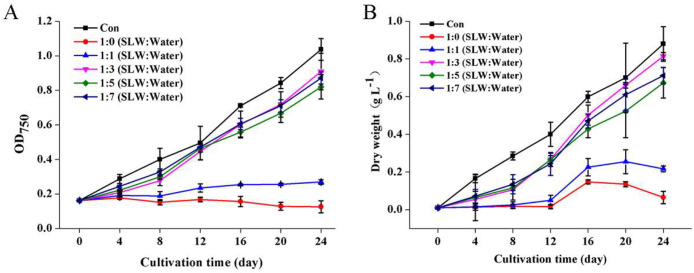
Cell growth (A) and biomass production (B) of *Vischeria* sp. WL1 cultivated in different SL^+^ waters. The SL water was diluted 2, 4, 6 and 8 folds, respectively, and was supplemented with additional NaNO_3_, K_2_HPO_4_ and EDTA-Fe^2+^_._ Con, BG11m medium. SLW, salt lake water. Values are the mean ± SD (*n* = 3).

Growth of *Vischeria* sp. WL1 in the diluted SL^+^ waters and BG11m medium (as a control) were evaluated during 24 days of cultivation (Fig. 1). As indicated by OD_750_ (Fig. 1A), *Vischeria* sp. WL1 could rapidly grow in the SL^+^ waters with 4, 6 and 8-fold dilutions, while its growth was severely inhibited in the non-diluted and 2-folds diluted SL^+^ waters. Biomass concentration showed a similar changing trend (Fig. 1B). On the tested last day (day 24), the biomass concentration of *Vischeria* sp. WL1 cultivated in the 4-folds diluted SL^+^ water (0.82 g L^−1^) was not significantly different from that in the control (0.88 g L^−1^), while the biomass concentrations in the SL^+^ waters with 6 and 8-fold dilutions were significantly lower (nearly 20 % reduction) than that in the control. Thus, the 4-folds diluted SL^+^ water is an optimum cultural medium for biomass production of *Vischeria* sp. WL1.

### 3.2 Effects on cell size and pigment contents

**Fig. 2:**
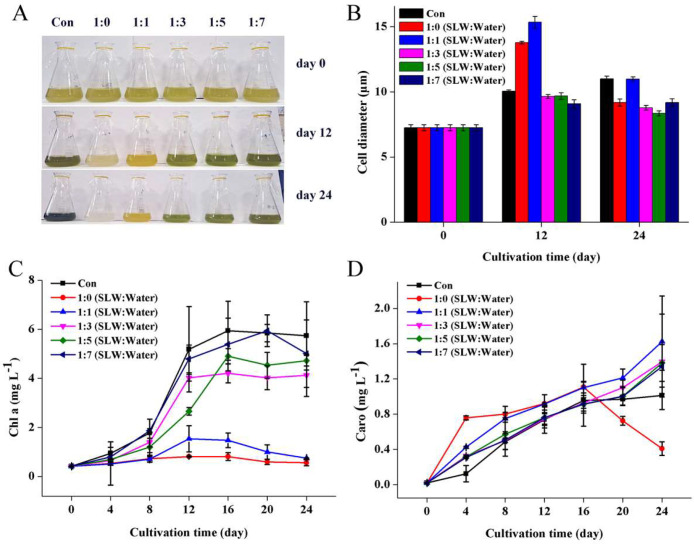
Color changes of the *Vischeria* sp. WL1 cultures (A) and characterization of cell size (B), Chl a (C) and carotenoid (D) contents in the cultures. Values are the mean ± SD (for panel B, *n* = 100; for panels C and D, *n* = 3).

The effects of the diluted SL^+^ waters on the morphology (cell size) and photosynthetic pigments (Chl a and carotenoids) contents of *Vischeria* sp. WL1 were further evaluated (Fig. 2). The color changes of the cultures were shown in Fig. 2A. The average cell size was obviously enlarged on day 12 when *Vischeria* sp. WL1 was cultivated in the non-diluted and 2-folds diluted SL^+^ waters, compared to the control; on day 24, except in the 2-folds diluted SL^+^ water, the average cell sizes were decreased in other diluted SL^+^ waters (Fig. 2B; Supplemental Figs. S1–S4). Overall, the Chl a contents of the cultures in SL^+^ waters were lower than that of the control (Fig. 2C). Particularly, the growth of *Vischeria* sp. WL1 (in terms of Chl a content) was severely inhibited in the non-diluted and 2-folds diluted SL^+^ waters. In other diluted SL^+^ waters, the Chl a contents were not obviously increased from day 12 or day 16 (Fig. 2C), which is unlike the changes of biomass concentration (Fig. 1B). Similar phenomenon was also observed in the green microalga *Tetraselmis suecica* cultivated under salinity stress [34]. It suggests that biomass increase is sometimes not synchronous with the increase of Chl a content under abiotic stress. Except the non-diluted SL^+^ water, the carotenoid contents in the diluted SL^+^ waters showed a continuous increase; the 2-folds diluted SL^+^ water resulted in a relatively higher carotenoid content (Fig. 2D). These results demonstrate the potentially adaptive changes of *Vischeria* sp. WL1 cells under the stressful salinity conditions.

### 3.3 Oil production and analysis of fatty acid composition

**Fig. 3:**
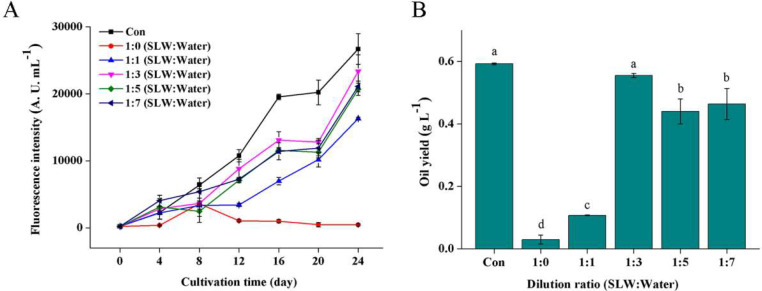
Changes in Nile red-stained oil contents of *Vischeria* sp. WL1 cultures (A) and the oil production on day 24 (B) in different SL^+^ waters. Values are the mean ± SD (*n* = 3). Different letters (a, b, c or d) above the columns indicate significant difference between the groups (*P* < 0.05).

The effects of diluted SL^+^ waters on oil production of *Vischeria* sp. WL1 were also evaluated during 24 days of cultivation. The fluorescent staining with Nile red offers a rapid and cost-effective analysis tool for quantifying neutral oil content [29]. The changes in total oil content of the cultures were detected by Nile red staining (Fig. 3A; Supplemental Fig. S5). The oil production was continuously increased along with the extension of cultivation time except in the non-diluted water. The absolute quantification of oils showed that the culture in the 4-folds diluted SL^+^ water achieved an oil yield of 0.56 g L^−1^, not significantly lower than that in the control (Fig. 3B). In our previous study, the control (BG11m medium) was found to be most favorable for biomass and oil production in *Vischeria* sp. WL1 [8]. The cultures in the 6- and 8-folds diluted SL^+^ waters achieved relatively lower oil yields (0.47 and 0.45 g L^−1^, respectively) (Fig. 3B). Similar to biomass production, both the non-diluted and 2-folds diluted SL^+^ waters were found to be unsuitable for oil production (Fig. 3B). These results indicate that the SL^+^ water with a 4-fold dilution is most favorable for oil production of *Vischeria* sp. WL1.

**Fig. 4:**
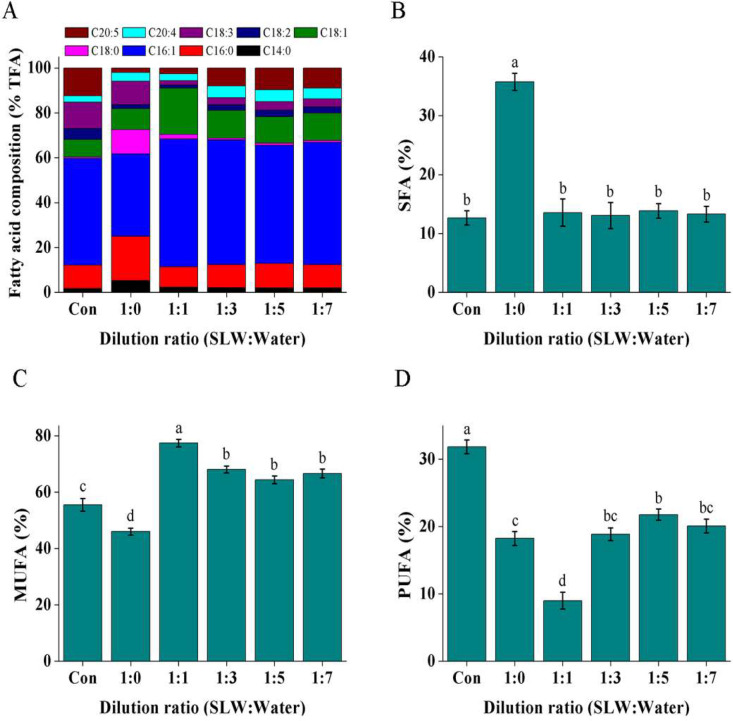
The fatty acid compositions (A) and SFA (B), MUFA (C) and PUFA (D) contents of the oils harvested from *Vischeria* sp. WL1 cultures in different SL^+^ waters. Values are the mean ± SD (*n* = 3). Different letters (a, b, c or d) above the columns indicate significant difference between the groups (*P* < 0.05).

The fatty acid compositions of the oils harvested from the cultures on day 24 were further analyzed (Fig. 4). Among the oils, the oil of the culture from the 2-folds diluted SL^+^ water contained the largest proportion (20.6 %) of C18:1 fatty acid (Fig. 4A). However, as described above, this cultural condition is not suitable for oil production. The oils of the cultures from 4-, 6- and 8-folds diluted SL^+^waters also contained relatively high C18:1 fatty acid proportions (11.7∼12.4 %), higher than that (8.0 %) in the control; however, the eicosapentaenoic acid (C20:5) proportion varied from 8.0 % to 9.7 % in the three cultures, lower than that (12.4 %) in the control (Fig. 4A). Moreover, total saturated fatty acid (SFA) proportions (13.1∼13.8 %) in the three cultures were not significantly different from that in control (Fig. 4B); total monounsaturated fatty acid (MUFA) proportions (64.4∼68.1 %) in them were higher than that in the control (55.5 %, Fig. 4C); total PUFA proportions (18.9∼21.8 %) in them were lower than that in the control (31.9 %, Fig. 4D). The microalgal oils with high contents of SFA and MUFA are the ideal feedstock for biodiesel production [35]. Thus, employing the diluted SL^+^ water to cultivate *Vischeria* sp. WL1 may serve as a cost-effective way to produce algal biodiesel.

### 3.4 Protein and starch accumulation

**Fig. 5:**
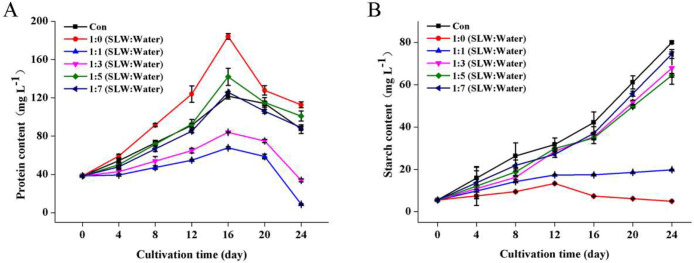
Changes of protein and starch contents of *Vischeria* sp. WL1 cultures in different SL^+^ waters. Values are the mean ± SD (*n* = 3).

In addition to oil, protein and starch are also accumulated and stored in microalgal cells, and their relative contents can be affected by cultural conditions [36,37]. To explore their changing profiles, protein and starch contents of the cultures were also investigated during cultivation (Fig. 5). In all of the cultures, the protein contents reached a highest point on day 16 and then gradually decreased (Fig. 5A). However, similar to the control, the starch contents were continuously increased in the cultures cultivated by 4-, 6- and 8-folds diluted SL^+^ waters (Fig. 5B). It implies that the photosynthetically fixed carbon source might be allocated from protein biosynthesis to oil and starch biosynthesis during the late phase of cultivation.

**Fig. 6:**
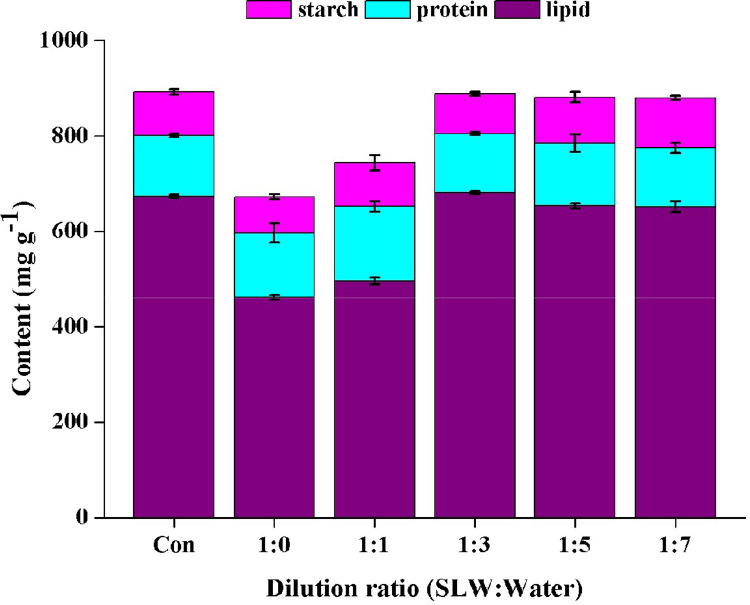
The compositional relationship of starch, protein and oil in dried *Vischeria* sp. WL1 cultures after 24 days of cultivation in different SL^+^ waters. Values are the mean ± SD (*n* = 3).

A comparison of oil, protein and starch contents in the biomass on day 24 (Fig. 6) showed their total contents were not much different among the control and the groups cultivated by 4-, 6- and 8-folds diluted SL^+^ waters. Also, the contents of oils were highest among the three molecules, ranging from 652.0 to 681.0 mg g^−1^. Many factors affect oil biosynthesis in microalgae, including nitrogen, phosphorus, carbon, metal ions and salinity [32], [33], [34], [35], [36], [37], [38]. The diluted Gouchi Salt Lake water was supplemented with additional nitrogen, phosphorus and iron nutrients for use. Thus, the above comparison further indicates the feasibility of diluted SL water with supply of the three nutrients for preferentially producing oil in *Vischeria* sp. WL1. Various sodium salts (NaCl, Na_2_S_2_O_3_ or NaHCO_3_) have been used to induce oil accumulation in microalgae with a two-phase cultivation mode, in order to cope with the contradiction between biomass increase and oil production [39]. In contrast, our study suggests that essential nutrients-augmented SL water can be utilized to cultivate microalgae for efficiently harvesting biomass and oil, avoiding the extra labor cost in adopting the two-phase cultivation mode.

As one of abiotic stress factors, salinity stress (usually high concentrations of NaCl) is widely used as an effective tool to enhance metabolite production in microalgae [40]. High salinity can induce ionic, osmotic and oxidative stresses on cells [41]. To minimize the resulting damage, microalgae have developed various coping mechanisms, including accumulation of oils and carbohydrates as storage molecules [41], [42], [43]. That should be the main reason that the Gouchi Salt Lake water could induce the oil production in *Vischeria* sp. WL1. An increased production of starch (carbohydrate) was also observed in this study. In addition, salinity stress can result in physiological, biochemical or morphological changes of microalgal cells [24,44]. The changes in cell size and pigment composition of *Vischeria* sp. WL1 were observed in this study. However, the composition of mineral ions in the SL water is very unbalanced compared to the widely used algal medium BG11. For example, despite being diluted 4–8 folds, the contents of chloride and sulfate in the SL water were still much higher than those in BG11m medium. In some studies, salt-rich (mainly NaCl) wastewaters were effectively utilized to cultivate microalgae for nutrient removal and biomass harvesting [45,46]. Differently, our study represents the effects of complex salinity ions from SL water on biomass and metabolite production in microalgae, which is more likely to the utilization of seawater in microalgae cultivation [14,15]. Therefore, this study also sheds some insights into the effects of complex salt-lake mineral ions on microalgal physiology and metabolism.

## 4 Conclusions

SL water is a precious resource awaiting full development in microalgae cultivation for harvesting biomass and value-added biochemicals. It provides sufficient mineral elements for microalgal growth as well as helps to save freshwater resource in the inland areas. In this study, we attempted to cultivate the terrestrial oil-producing strain *Vischeria* sp. WL1 with nutritionally augmented, diluted SL water. It was found that *Vischeria* sp. WL1 adapted well in the 4-, 6- and 8-folds diluted SL water with supplementation of additional nitrogen, phosphorus and iron. A 4-fold dilution was found to be most favorable for biomass increase and oil production in this strain. Under this condition, *Vischeria* sp. WL1 gained a biomass yield of 0.82 g L^−1^ and an oil yield of 0.56 g L^−1^ after 24 days of cultivation. The achieved biomass and oil production levels were comparable to the levels we previously established in the optimum medium BG11m. In addition, the cell size, starch content and protein content of *Vischeria* sp. WL1 were greatly affected during the cultivation. Mass cultivation of this strain under the outdoor environment near the Salt Lake remains to be explored in the future. In general, this study indicates that dilution of SL water and supplementation of necessary additional nutrients can serve as a cost-efficient strategy to save freshwater resource and mineral nutrient cost in microalgae cultivation. This strategy will assist the rapid development of algal production industry in the inland areas.

## CRediT authorship contribution statement

**Xiang Gao:** Conceptualization, Supervision, Writing – original draft. **Xin jing:** Data curation, Formal analysis, Investigation, Methodology, Writing – original draft. **Jiahong Li:** Data curation, Formal analysis, Investigation, Methodology. **Min Guo:** Formal analysis, Investigation, Methodology. **Lei Liu:** Data curation, Formal analysis, Investigation, Methodology. **Zhengke Li:** Validation, Writing – review & editing. **Kaihui Liu:** Validation, Writing – review & editing. **Derui Zhu:** Conceptualization, Funding acquisition, Supervision, Validation, Writing – review & editing.

## Declaration of Competing Interest

The authors declare that they have no known competing financial interests or personal relationships that could have appeared to influence the work reported in this paper.

## Data Availability

Data will be made available on request.

## Appendix Supplementary materials

Image, application 1

Image, application 2
