# Supplementary material for: Exploitation of inland salt lake water by dilution and nutrient enrichment to cultivate Vischeria sp. WL1 (Eustigmatophyceae) for biomass and oil production
Source: Biotechnol Rep (Amst). 2023 Dec 11;41:e00823. doi: 10.1016/j.btre.2023.e00823 (PMC10765011; doi:10.1016/j.btre.2023.e00823)
Supplement: Supplementary file 1 [file mmc1.docx]

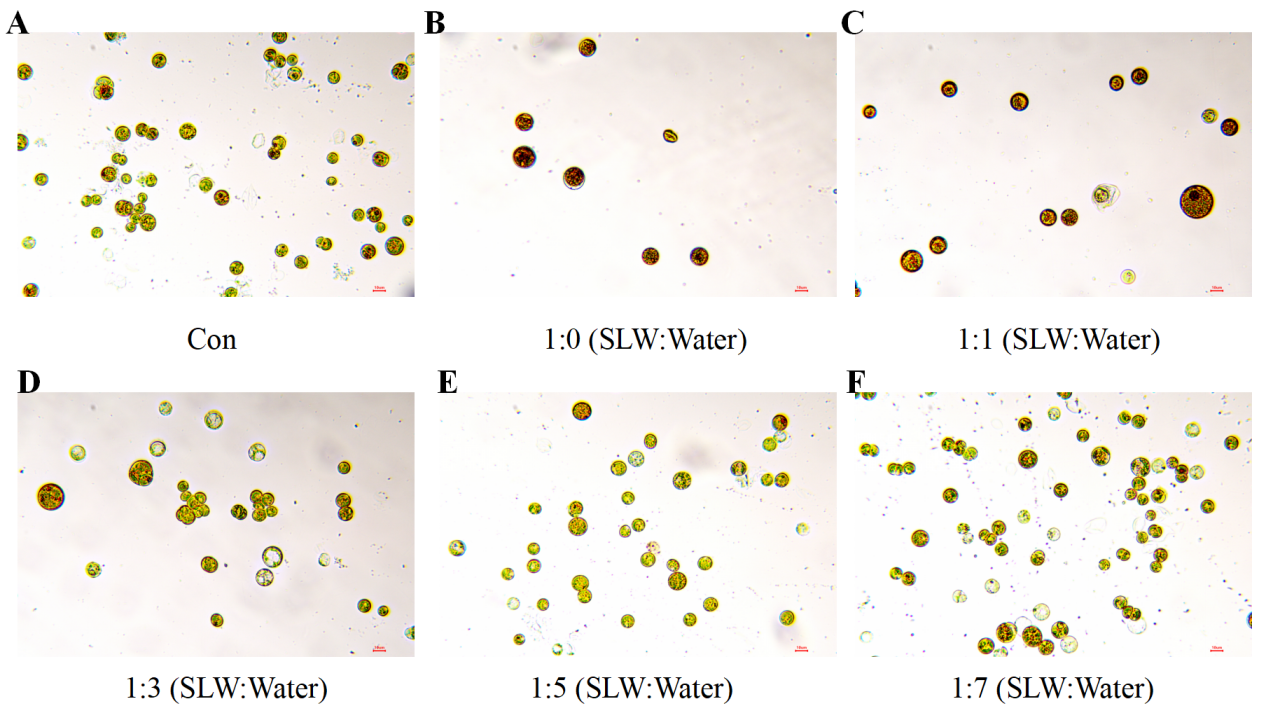


**Figure S1** Microscopic observation of *Vischeria* sp. WL1 cells after 12 days of cultivation in different SL^+^ waters. SLW, salt lake water. Bar, 10 μm.


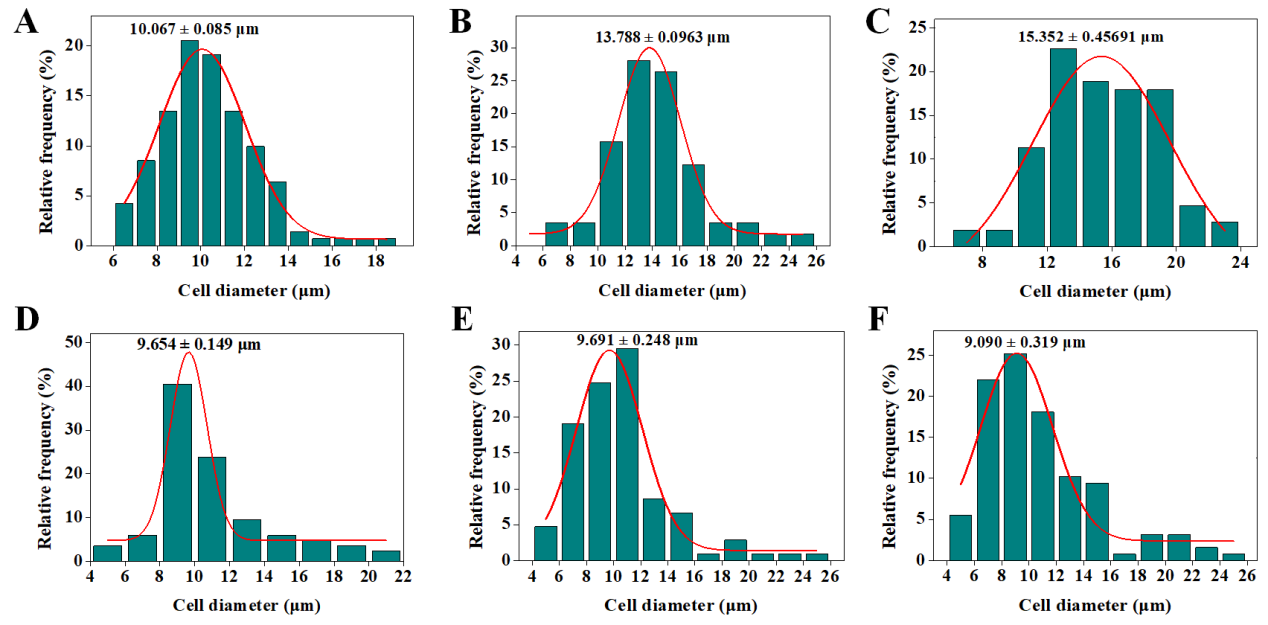


**Figure S2** Measurement of the cell size of *Vischeria* sp. WL1 after 12 days of cultivation in different SL^+^ waters. A, control (BG11m). B, SLW:water of 1:0. C, SLW:water of 1:1. D, SLW:water of 1:3. E, SLW:water of 1:5. F, SLW:water of 1:7. For each condition, 100 cells were randomly chosen for measurement.


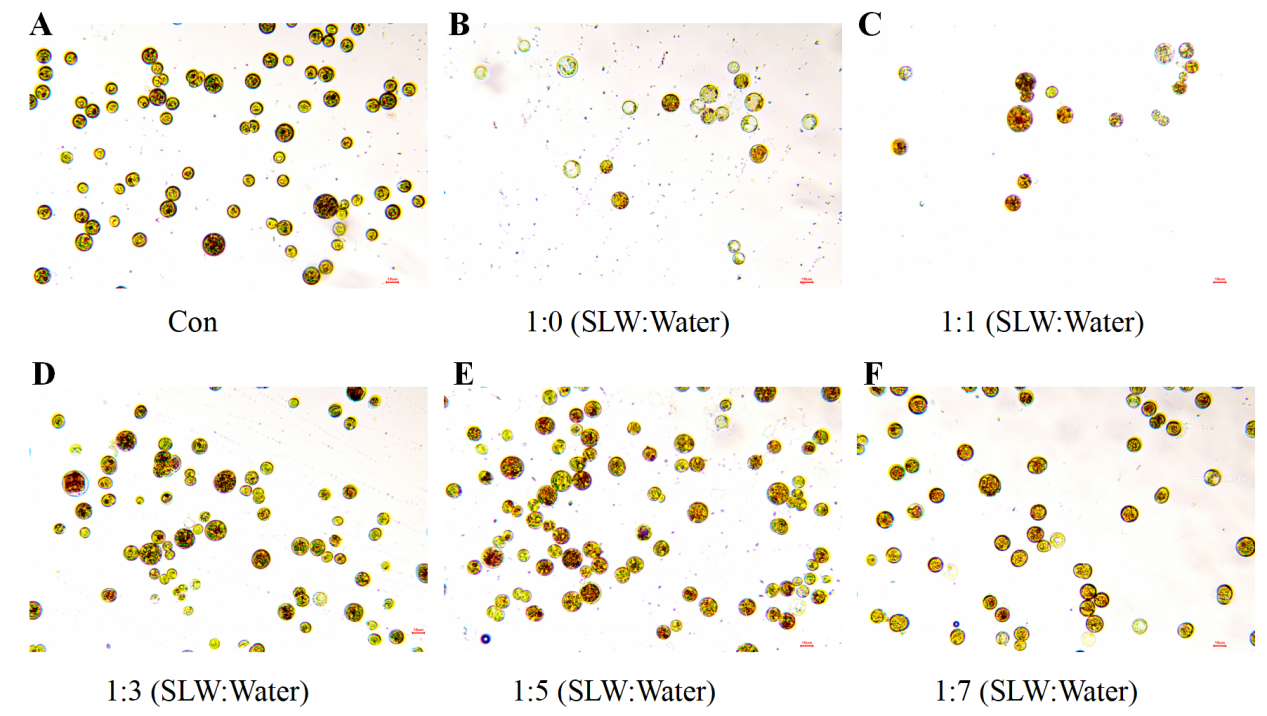


**Figure S3** Microscopic observation of *Vischeria* sp. WL1 cells after 24 days of cultivation in different SL^+^ waters. SLW, salt lake water. Bar, 10 μm.


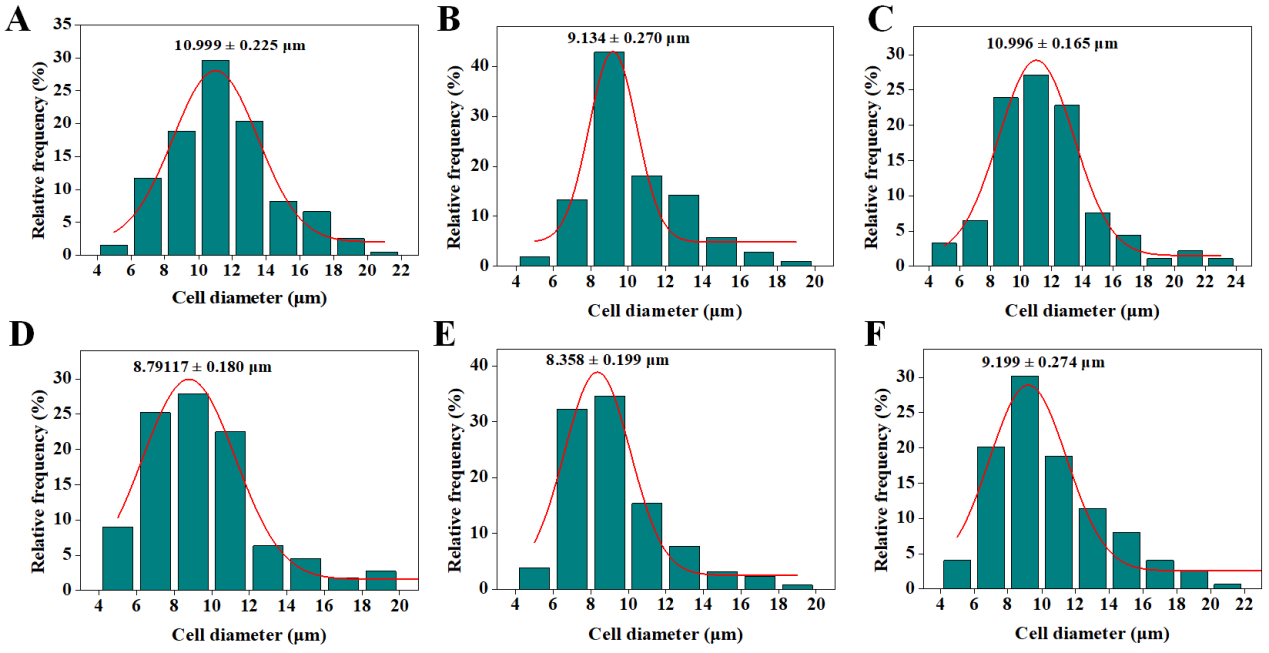


**Figure S4** Measurement of the cell size of *Vischeria* sp. WL1 after 24 days of cultivation in different SL^+^ waters. A, control (BG11m). B, SLW:water of 1:0. C, SLW:water of 1:1. D, SLW:water of 1:3. E, SLW:water of 1:5. F, SLW:water of 1:7. For each condition, 100 cells were randomly chosen for measurement.
