# Supplementary material for: Exploitation of inland salt lake water by dilution and nutrient enrichment to cultivate Vischeria sp. WL1 (Eustigmatophyceae) for biomass and oil production
Source: Biotechnol Rep (Amst). 2023 Dec 11;41:e00823. doi: 10.1016/j.btre.2023.e00823 (PMC10765011; doi:10.1016/j.btre.2023.e00823)
Supplement: Supplementary file 2 [file mmc2.docx]

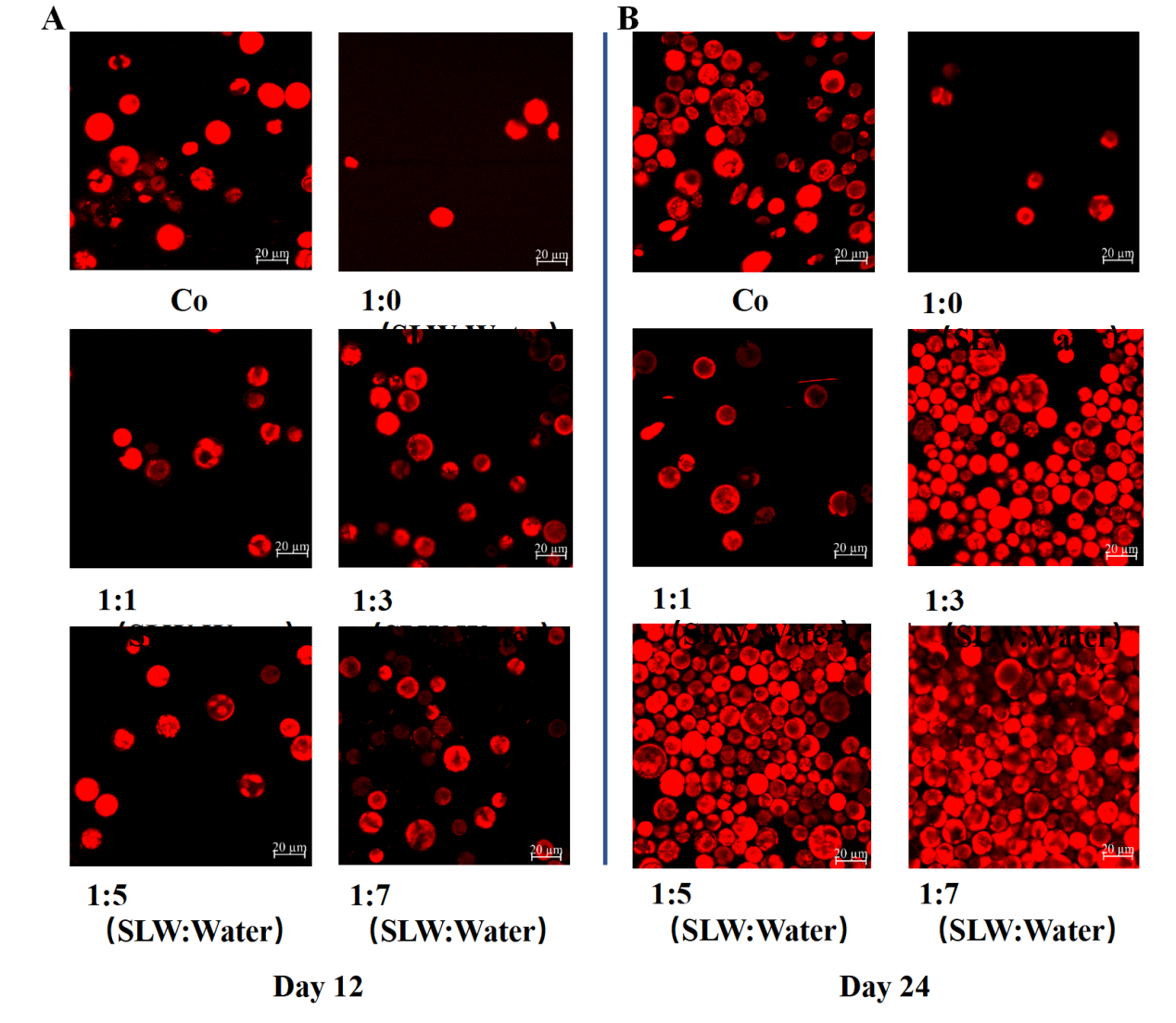


**Figure S5** Microscopic observation of Nile red-stained *Vischeria* sp. WL1 cells after 12 (A) and 24 (B) days of cultivation in different SL^+^ waters. SLW, salt lake water. Bar, 20 μm.
